# Chemical Fractionation Joint to In-Mixture NMR Analysis for Avoiding the Hepatotoxicity of *Teucrium chamaedrys* L. subsp. *chamaedrys*

**DOI:** 10.3390/biom11050690

**Published:** 2021-05-05

**Authors:** Simona Piccolella, Monica Scognamiglio, Brigida D’Abrosca, Assunta Esposito, Antonio Fiorentino, Severina Pacifico

**Affiliations:** Dipartimento di Scienze e Tecnologie Ambientali, Biologiche e Farmaceutiche, Università degli Studi della Campania “Luigi Vanvitelli”, Via Vivaldi 43, 81100 Caserta, Italy; simona.piccolella@unicampania.it (S.P.); monica.scognamiglio@unicampania.it (M.S.); brigida.dabrosca@unicampania.it (B.D.); assunta.esposito@unicampania.it (A.E.)

**Keywords:** *Teucrium chamaedrys* L. subsp. *chamaedrys*, NMR-based metabolomics, phenylethanoid glycosides, *neo*-clerodanes, antioxidant activity, cytotoxicity

## Abstract

Dietary supplements based on *Teucrium chamaedrys* L. subsp. *chamaedrys* aerial parts were banned, due to the hepatotoxicity of furan-containing *neo*-clerodane constituents. Indeed, the plant leaf content in phenolic compounds could be further exploited for their antioxidant capability. Accordingly, bio-guided fractionation strategies have been applied, obtaining seven partially purified extracts. These latter were chemically investigated through 1D and 2D NMR techniques and tested for their antiradical, reducing and cytotoxic capability. Data acquired highlighted that, through a simple phytochemical approach, a progressive *neo*-clerodane depletion occurred, while maximizing phenylethanoid glycosides in alcoholic fractions. Thus, although the plant cannot be used as a botanical remedy as such, it is suggested as a source of healthy compounds, pure or in mixture, to be handled in pharmaceutical, nutraceutical and/or cosmeceutical sectors.

## 1. Introduction

Botanical medicine continues to grow as plant-based preparations are commonly perceived as natural sources of chemicals with healthy benefits. Indeed, the specious awareness “natural is safe” might hide adverse effects, related to chemical compounds potentially harmful to human health [1,2]. This is mainly true for furan-containing *neo*-clerodane diterpenoids [3], such as teucrin A and teuchamaedryn A, which are causes of liver failure, so much so that *Teucrium* spp., especially *Teucrium chamaedrys*, are prohibited to be used [4].

*Teucrium chamaedrys*, also known as wall germander, is an evergreen small shrub, native to the Mediterranean region, whose employment for curative purposes is traced back to ancient times. The leaves are reported to exhibit antioxidant, anti-inflammatory, antirheumatic, carminative, diaphoretic, astringent, digestive, stimulant, laxative, aromatic, diuretic and tonic activity [5,6]. Based on its properties, wall germander was used as spice, for imparting the aromatic flavor to base wine for vermouth or other liqueurs production [7], and to prepare, starting from the mid-1980s of the last century, herbal tea bags, or germander-containing capsules for weight loss [8]. Unfortunately, more than fifty hepatotoxicity reports (mainly hepatitis and liver cirrhosis), including a sudden death case, were recorded following wall germander-based supplementation [9,10]. For this reason, French Pharmacovigilance Authorities in 1992 forced its withdrawal from the market, and some years later the Italian Ministry of Health considered wall germander flowering tops as poison, as well as all the preparations obtained from it (powder, extract, tincture, etc.).

The heterocyclic aromatic moiety of the furan-containing *neo*-clerodane diterpenoids undergoes oxidation by CYP3A4 to achieve reactive epoxides [11]. These latter were observed to cause cell disruption in animal models, through covalent binding to cell proteins, hepatic glutathione depletion, mitochondrial permeability transitions, and cytoskeleton membrane injury. Cell culture studies highlighted the hepatocyte apoptosis-inducing effect of the reactive epoxide. Furthermore, epoxide hydrolase on plasma membranes was found as a target of antibodies in the sera of patients who drank germander teas for 2–3 months [12].

However, the use of alcoholic extracts of the plant is still allowed as minor constituent in stomachic liqueur preparations for their flavoring and bittering features, also based on the opinion of the Scientific Committee on Food, establishing that teucrin A content in alcoholic beverages is almost 6 times lower than that present in the hepatotoxic germander capsules [13].

Indeed, other secondary metabolites, mainly iridoids and phenylethanoid glycosides [14,15], are also abundant in *T. chamaedrys* aerial parts, and their recovery needs to be pursued, while avoiding *neo*-clerodane toxicity. Hepatoprotective, antioxidant activity, anti-inflammatory and α-glucosidase inhibitory activities are ascribed to phenylethanoid glycosides, which also appeared to positively prevent dopaminergic neuronal damage, β-amyloid induced neurotoxicity, and to slow down the secretion of proinflammatory cytokines in autoimmune hepatitis animal models [16]. Thus, in order to enhance the exploitation of antioxidant compounds from wall germander, a fractionation strategy was suggested in a bio-guided scenario. Antiradical and reducing tests were applied to the alcoholic extract, as well as to the fractions derived, which were metabolically profiled by 1D and 2D NMR techniques. Cytotoxic effects were also assessed towards human hepatoblastoma HepG2 cells. Furthermore, as anticancer effects of *Teucrium* species were observed in lung carcinoma COR-L23, non-small-cell lung cancer H322 and A549 [17,18], and against breast adenocarcinoma MDA-MB-361 cells [19], the cytotoxicity evaluation of the constituted fractions was carried out on two other cancer cells, including A549 cells and, according to ethnobotanical use of *Teucrium chamaedrys* L. for the treatment of uterus infections [20], human cervix adenocarcinoma HeLa cells.

## 2. Materials and Methods

### 2.1. Materials

All of the solvents and reagents used for assessing antioxidant screening were purchased from Sigma-Aldrich Chemie (Buchs, Switzerland) except ABTS, which was bought from Roche Diagnostics (Roche Diagnostics, Mannheim, Germany). Cell culture medium and reagents for cytotoxicity testing were purchased from Invitrogen (Paisley, UK); MTT [3-(4,5-dimethyl-2-thiazolyl)-2,5-diphenyl-2H-tetrazolium bromide] was bought from Sigma-Aldrich Chemie. Deuterated solvents and internal standard for NMR-based metabolic profiling analyses were purchased from Sigma-Aldrich Chemie.

Leaves belonging to the species *T. chamaedrys* L. subsp. *chamaedrys*, a small eurimediterranean shrub characteristic of the coastal macchia vegetation [21], were collected at the “Castel Volturno” Nature Reserve (Caserta, Italy). Voucher specimens (CE0037) have been deposited at the Herbarium of the Department of Environmental, Biological and Pharmaceutical Sciences and Technologies, University of Campania “Luigi Vanvitelli”.

### 2.2. Extraction and Fractionation Procedures

**Figure 1:**
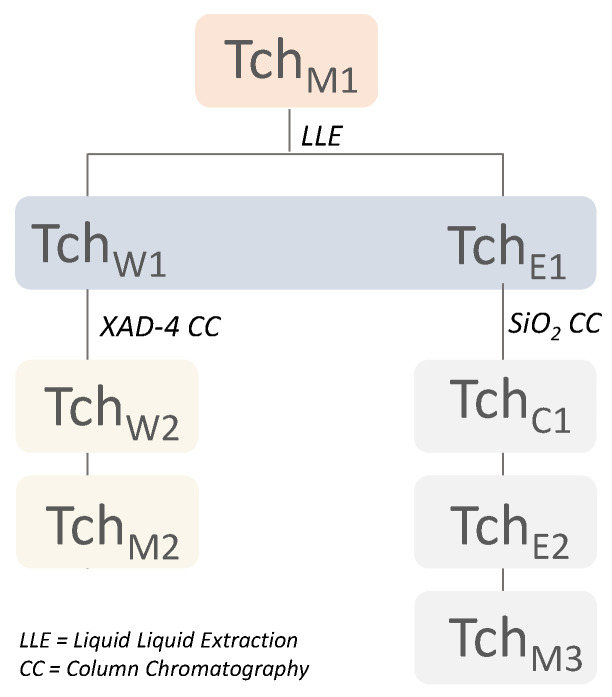
Fractionation scheme of *T. chamaedrys* leaf methanolic parental extract (Tch_M1_).

Leaves were dried in a hot-air convection oven at 45 °C for 5 days and then extracted by ultrasound-assisted maceration (UAM) (Branson Ultrasonics^TM^, Danbury, CT, USA) for 2 h, using MeOH as extracting solvent. The obtained crude extract (6.0 g, Tch_M1_), solubilized in water, underwent liquid–liquid extraction using ethyl acetate as extracting solvent. Thus, an aqueous (Tch_W1_) and an organic fraction (Tch_E1_) were obtained. The first one (5.0 g) was chromatographed using XAD-4 resin and H_2_O and MeOH as eluting solvents, yielding fractions Tch_W2_ (2.12 g) and Tch_M2_ (2.43 g). Instead, the organic fraction (Tch_E1_; 926.1 mg) was chromatographed by CC-SiO_2_, using, as eluents, three solvents with increasing polarity (CHCl_3_, EtOAc and MeOH), yielding three fractions: Tch_C1_ (333.4 mg), Tch_E2_ (161.0 mg) and Tch_M3_ (191.0 mg). The fractionation scheme is depicted in Figure 1.

### 2.3. NMR-Based Metabolic Profiling

Water and methanol fractions (40.0 mg each) were dissolved in 1.0 mL of a solvent system made up of K_2_HPO_4_ buffer (pH 6.0, 90 mM) in D_2_O (containing the internal standard) and CD_3_OD (1:1 *v*/*v*). Trimethylsilylpropionic-2,2,3,3-*d*_4_ acid sodium salt (TSP-*d*_4_, 0.1%, *w*/*v*) was used as internal standard. EtOAc fractions (40.0 mg) were dissolved in 1 mL of CD_3_OD, containing 0.1% of hexamethyldisilane as internal standard. The CHCl_3_ fraction (40.0 mg) was dissolved in 1 mL of CDCl_3_, containing 0.1% of hexamethyldisilane as internal standard, and 600 μL of each solution were analyzed by NMR.

NMR spectra were recorded at 25 °C on a Varian Mercury Plus 300 Fourier transform NMR at 300.03 MHz for ^1^H and at 75.45 MHz for ^13^C. Spectra were calibrated by setting the TSP-*d*_4_ peak at 0.00 ppm. Data acquisition parameters, for the ^1^H NMR spectrum, were as follows: 0.16 Hz/point, acquisition time (AQ) = 1.3 s, number of scans (NS) = 256, relaxation delay (RD) = 1.5 s, 90 pulse width (PW) = 6.6 μs, receiver gain = 22, number of data points (NP) = 4096, spectral width = 3065 Hz. A presaturation sequence was used to suppress the residual H_2_O signal. Line broadening of 0.3 Hz and zero-filling to 64 K were applied prior to Fourier transform. FIDs were Fourier-transformed, and the resulting spectra were manually phased and baseline-corrected using an ^1^H NMR processor (ACDLABS 12.0).

Heteronuclear single-quantum coherence (HSQC), and heteronuclear multiple-bond correlation (HMBC) spectra were recorded. They were performed with a 1.0 s relaxation delay and 3065 Hz spectral width in F2 and 18,116 Hz in F1. Qsine (SSB = 2.0) was used for the HMBC window function. The optimized coupling constants were ^1^J_HC_ = 140 Hz for HSQC and ^n^J_HC_ = 8 Hz for HMBC. COSY spectra were acquired with a 1.0 s relaxation delay and 2514 Hz spectral width in both dimensions. The window function for COSY spectra was sine-bell (SSB = 0).

Compound identity was confirmed by extensive 2D NMR analysis. When possible, data were compared with spectra of pure compounds, and/or with data available in literature. The confidence level for the identification of each metabolite is indicated by the superscript numbers (1–4) reported in Table 1 after the name of each compound, in accordance with the indication for the minimum reporting standards in metabolomics [22]. Briefly, four confidence levels were considered, as follows: 1. unequivocally identified compound (i.e., complete characterization through 1 and 2D NMR analyses and comparison with the spectrum of pure standards); 2. putatively identified compound (i.e., diagnostic signals or correlations detected in the extracts); 3. putatively identified compound class (i.e., either a portion of the molecule was not identified or some crucial correlations were missing to unequivocally identify the compound); 4. unknown compound (i.e., it was not possible to propose a structure for the detected signals). All of the identified compounds were previously reported from the plant species in object.

### 2.4. Antioxidant Efficacy Assessment

#### 2.4.1. DPPH^•^ Scavenging Capacity

DPPH^•^ scavenging capacity was evaluated by dissolving the crude extract/fractions under study in a DPPH^•^ methanol solution (9.4 × 10^−5^ M), in order to test different final concentration levels (5.0, 10.0, 25.0 and 50.0 μg mL^−1^). After stirring the reaction mixtures at room temperature for 30 min, 300 µL of each sample were transferred into a 96-well plate, and the absorbance was read at 520 nm using a Wallac Victor3 multilabel plate reader (PerkinElmer Inc., Waltham, MA). The results were expressed as previously reported [23].

#### 2.4.2. ABTS^•+^ Scavenging Capacity

ABTS^•+^ assay was performed according to [23]. All the investigated samples (5.0, 10.0, 25.0 and 50.0 μg mL^−1^, final concentration levels) were dissolved in 1.0 mL of the radical diluted solution. After 10 min, the absorbance was read at 734 nm using a Wallac Victor3 multilabel plate reader. The results were expressed as previously reported [23].

#### 2.4.3. Determination of oxygen radical absorbance capacity (ORAC)

In the ORAC assay, the investigated crude extract/fractions (20 µL; 5.0, 10.0, 25.0 and 50.0 μg mL^−1^, final concentrations) and fluorescein (120 µL; 70.0 nM, final concentration) solutions were preincubated for 15 min at 37 °C in 75.0 mM phosphate buffer (pH 7.4). Then, 2,20-azobis-(2-amidinopropane)-dihydrochloride (AAPH) solution (60.0 μL, 12.0 mM, final concentration) was rapidly added. In parallel with the samples, a blank (FL + AAPH) and solutions of the standard antioxidant Trolox^®^ (1–8 μM, final concentrations) were properly prepared in PBS. The fluorescence (λ_ex_ = 485 nm, λ_em_ = 525 nm) was recorded every 15 min for 120 min using a Wallac Victor3 (PerkinElmer, Waltham, MA, USA). Antioxidant curves (fluorescence versus time) were first normalized to the curve of the blank by multiplying original data by the factor fluorescence blank, t = 0/fluorescence sample, t = 0. From the normalized curves, the area under the fluorescence decay curve (AUC) was calculated as previously reported [24]. Linear regression equations between net AUC (AUC_antioxidant_ − AUC_blank_) and antioxidant concentration were calculated for all the samples. Antioxidant activity (ORAC value) was calculated using the Trolox^®^ calibration curve. The ORAC values are expressed as μmol Trolox^®^ equivalents.

#### 2.4.4. Determination of Mo(VI) Reducing Power

In order to carry out the quantitative determination of antioxidant capacity, investigated crude extract/fractions were dissolved in 1.0 mL of a reagent solution containing sulfuric acid (0.6 M), sodium phosphate (28.0 mM) and ammonium molybdate (4.0 mM). The samples were incubated at 95 °C for 90 min, and subsequently cooled down to r.t., and their absorption was measured at 675 nm by a Shimadzu UV-1700 spectrophotometer against the blank. The increase in absorption in reference to the blank estimates the reducing power [25].

### 2.5. Cell Culture and Cytotoxicity Evaluation

The human hepatoblastoma cell line (HepG2), lung epithelial cell line (A549) and cervical carcinoma cell line (HeLa) were purchased from ICLC (Interlab Cell Line Collection) at Istituto Nazionale per la Ricerca sul Cancro, Genoa (Genoa, Italy). HepG2 and HeLa cell lines were grown in RPMI (Invitrogen, Paisley, UK) containing 10% fetal bovine serum (Invitrogen, Paisley, UK), 50.0 U/mL of penicillin, and 100.0 μg/mL of streptomycin (Invitrogen, Paisley, UK), at 37 °C in a humidified atmosphere containing 5% CO_2_. The A549 cell line was plated and grown under the same conditions, except that DMEM high glucose (Invitrogen, Paisley, UK) was used instead of RPMI.

The cells were seeded in 96-multiwell plates at a density of 2.0 × 10^4^ cells/well. The day after, cells were treated with *T. chamaedrys* crude extract and fractions therefrom at three doses (25.0, 50.0, and 100.0 μg mL^−1^). After 24 and 48 h exposure times, MTT assay was performed, as previously described [26], and vinblastine (0.01 μM) served as a positive control [24].

### 2.6. Statistical Analysis

Antioxidant activity tests were carried out performing three replicate measurements for three samples (n = 3) of the crude extract and fractions therefrom (in total, 3 × 3 measurements). MTT assay was carried out performing twelve replicate (n = 12) measurements for three samples of the crude extract and fractions therefrom (in total: 12 × 3 measurements). All data were expressed as mean values ± standard deviation (SD). Student’s *t*-test was applied in order to determine statistical significance (significance level was set at *p*-value < 0.05). Pearson’s correlation coefficient was used to determine the relation between the variables. Antioxidant capacity and cell viability inhibition results were analyzed by principal component analysis (PCA). Before transforming the original measure variables into new variables (F), the variables were standardized to a mean of 0 and variance of 1. All analyses were performed with the GraphPad Prism 9.1.0 software package.

## 3. Results

### 3.1. NMR Metabolic Profile of Teucrium Chamaedrys L. subsp. Chamaedrys Leaf MeOH Extract and Fractions Therefrom

**Figure 2:**
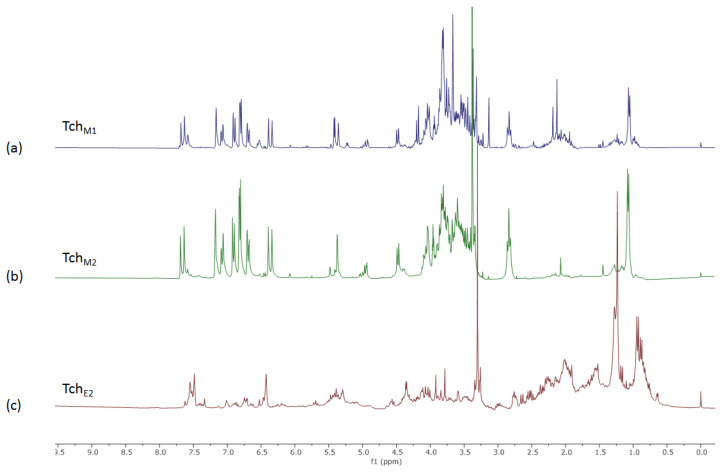
NMR profile of: (**a**) Tch_M1_ extract; (**b**) Tch_M2_ and (**c**) Tch_E2_ fractions.

The NMR profile of the Tch_M1_ extract, obtained by UAM in MeOH of *T. chamaedrys* leaves, is shown in Figure 2.

**Figure 3:**
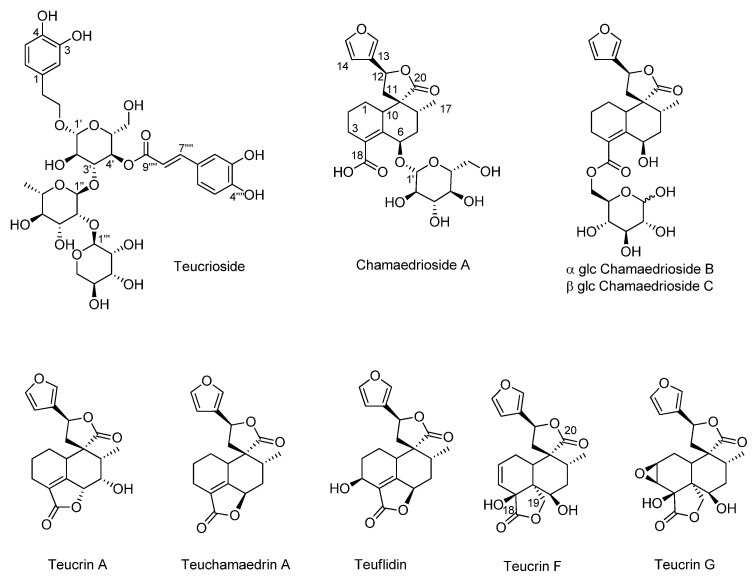
Chemical structures of the main constituents in Tch_M1_ extract, and fractions therefrom.

Literature data along with 2D-NMR experiments allowed the identification of the main constituents of the Tch_M1_ extract (Figure 3). The ^1^H NMR spectrum showed characteristic signals of teucrioside (Figure 2a) and *neo*-clerodanes diterpenes, previously reported from this species and as main components of the leaf extract [27]. Indeed, the spectrum showed signals consistent with a *trans*-caffeoyl moiety, with the catechol ring protons resonating as a *meta* coupled doublet at δ 7.14 (*J* = 1.8 Hz, H-2), a double doublet at δ 7.06 (*J* = 1.8 and 8.1 Hz, H-6) and an *ortho* coupled doublet at δ 6.88 (*J* = 8.1 Hz, H-5). Furthermore, the AB system for two olefinic protons at δ 7.65 (*J* = 15.9 Hz, H-7) and δ 6.34 (*J* = 15.9 Hz, H-8) was distinguishable. In the same region, signals of the catechol unit of the 3,4-dihydroxyphenyethanoylic moiety were evident as a *meta* coupled doublet at δ 6.79 (*J* = 2.1 Hz, H-2′), a double doublet at δ 6.67 (*J* = 2.1 and 7.8 Hz, H-6′) and an *ortho* coupled doublet at δ 6.78 (*J* = 7.8 Hz, H-5′). In the upfield region of the spectrum, the proton H-7′ of the ethanoyl chain at δ 2.83 was detected as triplet. The resonances attributable to the anomeric protons of two saccharide units were clear at δ 5.34 and 4.40 (*J* = 8.1 Hz), corresponding to an α-rhamnose and a β-glucose, respectively. The rhamnose presence was supported also by a doublet at δ 1.03 (*J* = 6.3 Hz). The anomeric signal of the third sugar unit, lyxose [28], was not detected because the resonance was too close to that of water and was therefore affected by the presaturation. However, its presence was confirmed by 2D-NMR analysis of the fractions obtained by the partial purification of the Tch_M1_ extract, which was subjected to liquid/liquid extraction (to give Tch_W1_ and Tch_E1_ fractions).

The Tch_W1_ fraction was further purified on Amberlite^®^ XAD-4 eluting first with water (Tch_W2_) and then with methanol (Tch_M2_). The signals belonging to the putative teucrioside were detected in the Tch_W1_ and Tch_M2_ fractions. The identity of the compound was proven thanks to 2D NMR analysis of the latest fraction. Indeed, all the HSQC, COSY and HMBC correlations were in accordance with the structure of the *trans*-caffeoyl and hydroxytyrosol moieties. Furthermore, also the presence of three sugar units was confirmed and the diagnostic correlations were evidenced.

In particular, the long-range correlation between the anomeric proton of glucose at δ 4.40 with the C-7 carbon of the hydroxytyrosol moiety, the correlation between the proton H-4′ of glucose (δ 4.94) and the C-9⁗ carbon on the caffeoyl moiety at δ 167.0, the correlation of the anomeric proton of rhamnose with the C-3 of glucose at δ 80.5, and finally the correlation of the anomeric proton of lyxose with the C-2″ carbon of rhamnose allowed to identify the linkage sites. While teucrioside was the main component of the Tch_M2_ fraction (Figure 2b), the parent fraction also contained signals belonging to primary metabolites (Table 1), all finally detected in the Tch_W2_ fraction and identified based on the comparison of NMR data to those reported in the literature [29]. The other main constituents of the extract were the *neo*-clerodane diterpenes. Hints for their presence in the Tch_M1_ extract were obtained by the characteristic signals of a β-substituted furan ring as a signal at δ 7.63 (H-16) and two multiplets at δ 7.57 (H-15) and 6.52 (H-14). These signals, along with minor signals still belonging to phenylethanoid glycosides, were detected in the Tch_E1_ fraction, which was further purified on silica gel to obtain three fractions: Tch_C1,_ Tch_E2_, Tch_M3_. While the methanol fraction (Tch_M3_) showed mainly the signals for teucrioside, both Tch_C1_ and Tch_E2_ contained signals attributable to clerodane diterpenes.

The aliphatic region of the spectrum of the Tch_C1_ fraction allowed us to assume the presence of terpene molecules and fatty acids. The multiplet at δ 2.20, attributable to the methylene group in α to the carboxyl carbon, the signal at δ 2.03, characteristic of the allyl protons in all unsaturated fatty acids, and the signal at δ 1.66 due to the β-methylene to carboxyl group were all diagnostic of fatty acids. The signal at δ 5.15 suggested the presence of olefinic protons of unsaturated acyl chains. The intense signal at δ 1.29 was related to all the saturated methylene groups. The aromatic region of the ^1^H NMR spectrum of the Tch_C1_ fraction showed signals due to the furan ring protons of *neo*-clerodanes. In the region of the protons geminal to oxygen functions, signals at δ 5.40 were attributable to the proton H-12 of *neo*-clerodanes with a five-membered spiro-lactone ring. Due to their relatively low abundance compared to the other components of the spectrum, it was not possible to definitively identify these compounds.

The ^1^H NMR spectrum of the Tch_E2_ extract highlighted the massive presence of *neo*-clerodanes (Figure 2c). In fact, once again, in the downfield spectral region the furan ring protons belonging to these compounds were evident. The identities were confirmed by 2D NMR analysis of this fraction. In particular, signals belonging to the β-substituted furan ring as at δ 7.57 (H-16), 7.46 (H-15) and 7.43 (H-14) showed HSQC correlations with the carbons at δ 140.6 (C-16), 144.1 (C-15) and 107.7 (C-14), respectively. Long-range correlations with the C-14 and C-16 carbon, as well as with the C-13 (δ 125.2), were also shown by the H-12 proton resonating either at δ 5.44 or at δ 5.69, depending on the substitution pattern of the *neo*-clerodane diterpenes generating the signal

The H-12 resonating at δ 5.44 showed HSQC correlations with the carbon at δ 72.1, in turn correlated in the HMBC to the protons at δ 2.32 and 2.61 (H-11). These protons showed further long-range correlations with the carbons at δ 51.0 (C-9), 42.2 (C-10) and 36.3 (C-8). A methyl signal at δ 1.17 (H-17), correlating in the HMBC with the C-9, also showed a COSY correlation with the proton at δ 1.89, which in turn correlated in the same experiment with the proton at δ 5.62 (H-7) bound to the carbon at δ 71.4 (C-7). These correlations therefore suggested the presence of teucrin A, reported as one of the main *neo*-clerodane diterpenoid compounds in *T. chamaedrys* [30].

The H-12 resonating at δ 5.69 showed HSQC correlations with the carbon at δ 74.9 (C-12) and COSY correlations to the protons at δ 2.65 and δ 2.34. These protons, bound to a carbon at δ 39.5, showed long-range correlations with the carbons at δ 181.9 (C-20), 56.1 (C-9), 41.5 (C10), 38.1 (C-8), and finally with the C-12. A methyl signal at δ 0.93 (H-17) showed long-range correlation with the C-8 and C-9 and a further correlation with a carbon at δ 71.9, which was bound to a proton at δ 5.18. However, this carbinol was not located at C-7 as in the case of teucrin A. Indeed, the H-17 proton was correlated in a COSY experiment with the proton at 2.16 (H-8), in turn correlating to the protons at δ 1.21 and 1.64 (H-7). Therefore, the carbon at δ 71.9 could be located at the C6. The H-7 protons showed HMBC correlations with the carbons at δ 139.8 (C-4), 142.2 (C-5), and 185.0 (C-18). This compound was hence putatively identified as teuchamaedrin A, reported together with the previously identified teucrin A, as the main responsible for the toxicity of the *T. chamaedrys* drug [30].

The methyl region showed many other signals putatively belonging to *neo*-clerodane diterpenes. A previous phytochemical study of *T. chamaedrys* L. subsp. *chamaedrys* leaf collected in the same study area also led to the isolation and identification of teucrin G and F, chamaedroxide, teuflidin and chamaedryosides A-C [31,32]. While it was not possible to definitively elucidate these compounds in the extracts, the H-17 methyl signals chemical shifts and the long-range correlations detected could suggest their presence.

### 3.2. Bioactivity of Teucrium chamaedrys L. subsp. Chamaedrys leaf MeOH Extract and Fractions Therefrom

#### 3.2.1. Antioxidant Capacity

The evaluation of the antioxidant capacity has been carried out using separate methods, due to the absence of a single valid method for the determination of the total antioxidant capacity of a system at present.

**Figure 4:**
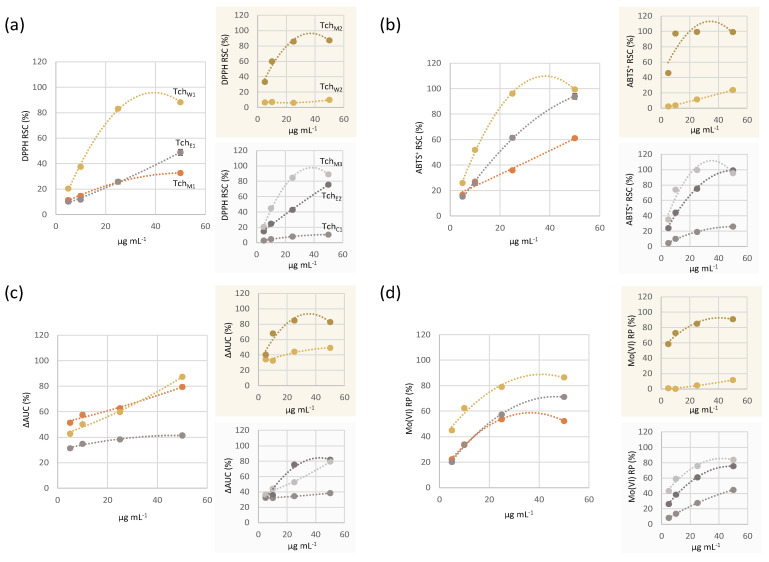
Antioxidant capacity (AC %) of •Tch_M1_ and of all the extracts deriving from its phytochemical fractionation: •Tch_W1_ (•Tch_W2_, •Tch_M2_) and •Tch_E1_ (•Tch_C1_, •Tch_E2_, •Tch_M3_), estimated as (**a**) DPPH RSC; (**b**) ABTS^+^ RSC; (**c**) ΔAUC; (**d**) Mo(VI) RP. Values are expressed as % (mean ± SD). (RSC = Radical Scavenging Capacity; AUC = Area Under Curve; RP = Reducing Power).

As depicted in Figure 4, the crude methanolic extract (Tch_M1_) showed a similar dose-response trend in the evaluation of radical scavenging capacity towards DPPH^•^ and ABTS^•+^. Indeed, the effectiveness appeared strongly dependent on the tested dose, where the latter probe was more responsible. In fact, Tch_M1_ determined an absorbance decrease in ABTS test, in which a 50% reduction of the radical oxidized form occurred as early as at 29.1 ± 0.7 µg mL^−1^ tested dose.

The ORAC assay measures the antioxidant inhibition of the peroxyl radical, induced by oxidation, reflecting the classic radical chain-breaking antioxidant activity by transfer of a hydrogen atom [33]. Briefly, the peroxyl radical reacts with fluorescein (FL, *marker*) to form a non-fluorescent product, which can be easily quantified by spectrofluorimetric measurements. The antioxidant capacity was determined by the decreasing rate and the loss of fluorescence, due to the oxidation of the marker over time [34]. ΔAUC values (AUC = Area Under Curve) could be calculated as the difference of areas below the decay curve of fluorescein in the tested sample and the blank (Figure 4). Moreover, their interpolation on the calibration curve of Trolox provided the concentration of Trolox having the same activity of the sample. Therefore, it has been estimated that in one gram of Tch_M1_ there are 1232,41 µmol equivalent of Trolox (ORAC Units).

The use of a molybdenum transition metal salt is a valid method in estimating the antioxidant capacity of the studied matrices. In fact, transition metals are particularly important catalysts of reactive oxygen species (ROS) production. The data obtained from the analysis of the Mo(VI) reducing power showed that Tch_M1_ was characterized by a good reducing power, strongly dependent also in this case by the sample tested dose, with an ID_50_ value estimated equal to 10.04 ± 0.9 µg mL^−1^.

When Tch_M1_ was fractionated, the further seven partially purified extracts obtained not only differed for polarity and solubility of their constituents but also in bioactivity. In particular, discontinuous liquid–liquid extraction provided the aqueous Tch_W1_, which exhibited a marked antiradical and reducing capacity, higher than the parental extract. In fact, it was able to reduce DPPH^•^ by 50% at a dose equal to 11.8 ± 0.5 µg mL^−1^, and the radical cation ABTS^•+^ at 9.5 ± 0.3 µg mL^−1^. This behavior was confirmed also in evaluating Mo(VI) reducing power, whose results showed a similar dose-response curve, but shifted at about 2-fold higher values for each tested concentration. This massive antioxidant activity could be explained considering that this fraction was enriched in phenylethanoid glycosides and traces of iridoid compounds, whereas *neo*-clerodanes were the main constituents of the organic counterpart Tch_E1_.

The further fractionation of Tch_W1_ by Amberlite^®^ XAD-4 resin column chromatography gave rise to the methanol Tch_M2_ fraction, whose constitution in active phenolic substances made it the most interesting antioxidant sample, as highlighted in all the performed tests (Figure 4).

#### 3.2.2. Cytotoxicity

**Figure 5:**
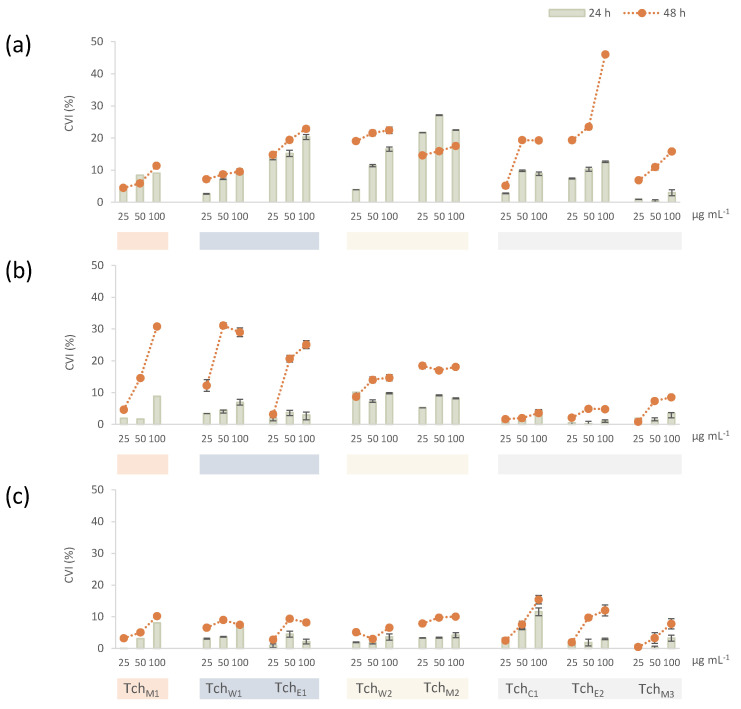
Cell viability inhibition (CVI%) of (**a**) HepG2, (**b**) HeLa and (**c**) A549 cell lines, treated with increasing doses of Tch_M1_ and of all the extracts deriving from its phytochemical fractionation: Tch_W1_ (Tch_W2_, Tch_M2_) and Tch_E1_ (Tch_C1_, Tch_E2_, Tch_M3_), estimated after 24 and 48 h exposure times. Values are expressed as % (mean ± SD).

The evaluation of cytotoxicity is a fundamental step in the definition of the potential harm that plant natural products could induce in the cells. The controversial hepatotoxicity of plants belonging to the *Teucrium* genus has long been attributed to *neo*-clerodane constituents. In this context, potential cytotoxic effects have been investigated towards human hepatocellular carcinoma cells HepG2, treated with increasing doses of leaf extract (25.0, 50.0 and 100.0 μg mL^−1^) and for two different exposure times (24 and 48 h). They represent a useful model for studying the function of CYP3A4, the enzyme responsible for the oxidation of the furan ring in highly toxic epoxy derivatives [35]. In order to highlight the potentially induced damage in other cell lines, the screening was extended to HeLa cervical cancer cells and A549 epithelial lung cancer cells. Cytotoxicity was estimated by MTT cell viability test, based on the intracellular reduction of tetrazolium salts in formazan crystals by mitochondrial dehydrogenases. Data acquired showed that the cytotoxic potential exerted by Tch_M1_ was different in the considered cell lines (Figure 5). In fact, although cell viability impairment appeared to be time-dependent and after 48 h also dose-dependent, HeLa cells proved to be the most sensitive to the treatment, in that their viability was inhibited by 30.8 ± 1.2% at the highest tested dose. HepG2 and A549 cell lines seemed to be not affected.

The cytotoxicity evaluation of fractions directly derived from the alcoholic parental extract showed that Tch_W1_ fraction did not contain any metabolites able to modify the cell viability rate. The absence of cytotoxic effects (CVI ≤ 10 %) also in this case was particularly evident in A549 and HepG2 cell lines, whereas HeLa viability decreased by about 30% after 48 h exposure time to the highest dose level. The lack of toxic effects on lung cancer cells was observed also in the treatment with Tch_E1_, whereas human cervix adenocarcinoma and hepatoblastoma cell growth was mildly influenced, probably due to the presence of *neo*-clerodane diterpenes. Indeed, HepG2 viability decreased by about 22.8 ± 0.7% after 48 h of treatment with the 100.0 µg mL^−1^ dose level (Figure 5). Accordingly, the enhanced amount of these metabolites in Tch_E2_ was responsible for a greater ability to inhibit cell viability in HepG2 cells. The cytotoxic effect, not detected in the other two cell lines, appeared to be strongly time- and dose-dependent. In fact, the highest cell viability inhibition, induced by the 100.0 µg mL^−1^ dose after 48 h exposure time, was 46.0 ± 0.5%. Moreover, it seems reasonable to assume that the additive effects of the polyphenolic components, constituting the Tch_M2_ and Tch_M3_ fractions, provided the detected beneficial effect.

### 3.3. Principal Component Analysis (PCA)

**Figure 6:**
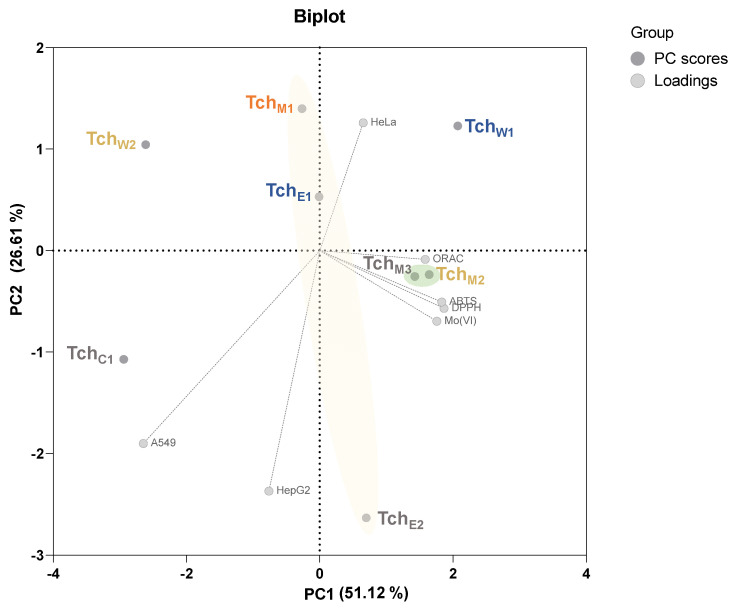
Principal component analysis (PCA) correlation biplot of investigated fractions, obtained using the data sets from antioxidant capability tests (DPPH, ABTS, ORAC and Mo(VI)RP) and CVI% on HepG2, HeLa and A549 cell lines.

Data obtained by principal component analysis (PCA) correlation biplot of investigated fractions, reported in Figure 6, summarize the main patterns of variation within the data sets from antioxidant capability tests (DPPH, ABTS, ORAC and Mo(VI)RP) and CVI% towards HepG2, HeLa and A549 cell lines. The different behavior of the studied fractions as antioxidant and/or cytotoxic samples were clearly highlighted, and it could be explained taking into account their peculiar enrichment in certain compound classes, due to polarity and solubility characteristics. In particular, the analyzed fractions are oriented along the first principal component (PC1), which attained 51.12% of variance, with an evident gradient produced by antioxidant data, whereas the second principal component (PC2) explained 26.61% of the total variation and was mainly related to cytotoxicity data. Thus, the two-dimensional graph was able to describe 77.73% of the variability in the experimental data. It is reasonable to assume that the depletion in the phenolic constituents from Tch_M1_ extract to the partially purified Tch_E2_ fraction is responsible for the enhanced CVI% observed, whereas the phenylethanoid glycosides-rich fractions (Tch_M2_ and Tch_M3_) are characterized by a marked scavenging and/or reducing capability. Finally, the Tch_W1_ sample, located in the upper right-hand side of the graph, was directly correlated with a good antioxidant activity and very low cytotoxic effects, which were mainly for HeLa cells. Furthermore, Tch_W2_ appeared inversely correlated to both biological activities. Tch_C1_, which was mainly constituted by diterpenes and unsaturated fatty acids, differed from all the other samples, in that its effectiveness was negatively correlated with antioxidant tests and exerted only poor effects on cell viability.

## 4. Conclusions

The metabolic complexity of *T. chamaedrys* subsp. *chamaedrys* leaf crude alcoholic extract was simplified through a bio-guided fractionation strategy. Fractions obtained were analyzed for their antioxidant capability, through four different assays, and cytotoxicity was evaluated towards three different cell lines. They mostly differed for their antioxidant capacity, and applying PCA, a better visualization of all results was attained. In fact, based on the PCA data, fractionation could play a stronger role in preserving the antioxidant activities of the wall germander leaves. The availability of fractions constituted by phenolic compounds as the main actors could give a renewed interest in wall germander.

Aerial parts of the plants could be used as a source of healthy compounds, pure or in mixture, to be handled in pharmaceutical, nutraceutical and/or cosmeceutical sectors.

## Figures and Tables

**Table 1:** Metabolites identified in *T. chamaedrys* L. subsp. *chamaedrys* fractions. The confidence level for the identification of each metabolite is indicated by the superscript numbers after the name of each compound (1–4, in accordance with the rules reported in the experimental section).

Compounds	Tch_W1_		Tch_E1_		
	Tch_W2_	Tch_M2_	Tch_C1_	Tch_E2_	Tch_M3_
Teucrioside ^1^		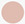			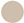
Other phenolic compounds ^4^					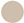
Teucrin A ^2^				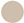	
Teuchamaedrin A ^2^				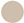	
Teucrin G ^3^				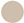	
Teucrin F ^3^				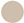	
Chamaedroxide ^3^				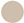	
Teuflidin ^3^				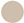	
Chamaedryosides A-C ^3^				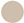	
Other *neo*-clerodane diterpenes ^4^			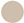	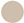	
Fatty acids ^3^			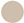		
Alanine ^2^	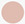				
Isoleucine ^2^	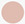				
Threonine ^2^	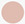				
Valine ^2^	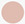				
Glucose ^2^	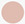				
Sucrose ^2^	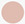				
Acetic acid ^2^	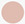				
Other organic acids ^4^	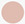				

## References

[1] Haslan H., Suhaimi F.H., Das S. (2015). Herbal supplements and hepatotoxicity: A short review. Nat. Prod. Commun..

[2] de Boer Y.S., Sherker A.H. (2017). Herbal and dietary supplement-induced liver Injury. Clin. Liver Dis..

[3] Cachet X., Langrand J., Riffault-Valois L., Bouzidi C., Colas C., Dugay A., Michel S., Boucaud-Maitre D. (2018). Clerodane furanoditerpenoids as the probable cause of toxic hepatitis induced by *Tinospora crispa*. Sci. Rep..

[4] Piozzi F., Bruno M., Ciriminna R., Fazio C., Vassallo N., Arnold N.A., de la Torre M.C., Rodriguez B. (1997). Putative hepatotoxic neoclerodane diterpenoids from *Teucrium* species. Planta Med..

[5] Gursoy N., Tepe B. (2009). Determination of the antimicrobial and antioxidative properties and total phenolics of two “endemic” Lamiaceae species from Turkey: *Ballota rotundifolia* L. and *Teucrium chamaedrys* C. Koch. Plant Foods Hum. Nutr..

[6] Vladimir-Knežević S., Blažeković B., Kindl M., Vladić J., Lower-Nedza A.D., Brantner A.H. (2014). Acetylcholinesterase inhibitory, antioxidant and phytochemical properties of selected medicinal plants of the Lamiaceae family. Molecules.

[7] Panesar P.S., Kumar N., Marwaha S.S., Joshi V.K. (2009). Vermouth production technology–an overview. Nat. Prod. Radiance.

[8] Herrera S., Bruguera M. (2008). Hepatotoxicidad inducida por el uso de hierbas y medicamentos para perder peso [Hepatotoxicity induced by herbs and medicines used to induce weight loss]. Gastroenterol. Hepatol..

[9] Gori L., Galluzzi P., Mascherini V., Gallo E., Lapi F., Menniti-Ippolito F., Raschetti R., Mugelli A., Vannacci A., Firenzuoli F. (2011). Two contemporary cases of hepatitis associated with *Teucrium chamaedrys* L. decoction use: Case reports and review of literature. Basic Clin. Pharmacol. Toxicol..

[10] Nencini C., Galluzzi P., Pippi F., Menchiari A., Micheli L. (2014). Hepatotoxicity of *Teucrium chamaedrys* L. decoction: Role of difference in the harvesting area and preparation method. Indian J. Pharmacol..

[11] Lekehal M., Pessayre D., Lereau J.M., Moulis C., Fouraste I., Fau D. (1996). Hepatotoxicity of the herbal medicine germander: Metabolic activation of its furano diterpenoids by cytochrome P450 3A Depletes cytoskeleton-associated protein thiols and forms plasma membrane blebs in rat hepatocytes. Hepatology.

[12] De Berardinis V., Moulis C., Maurice M., Beaune P., Pessayre D., Pompon D., Loeper J. (2000). Human microsomal epoxide hydrolase is the target of germander-induced autoantibodies on the surface of human hepatocytes. Mol. Pharmacol..

[13] European Commission Health & Consumer Protection Directorate General (2003). Opinion of the Scientific Committee on Food on Teucrin A, Major Component of Hydroalcoholic Extracts of Teucrium Chamaedrys (Wild Germander).

[14] Pacifico S., D’Abrosca B., Pascarella M.T., Letizia M., Uzzo P., Piscopo V., Fiorentino A. (2009). Antioxidant efficacy of iridoid and phenylethanoid glycosides from the medicinal plant *Teucrium chamaedrys* in cell-free systems. Bioorg. Med. Chem..

[15] Frezza C., Venditti A., Matrone G., Serafini I., Foddai S., Bianco A., Serafini M. (2018). Iridoid glycosides and polyphenolic compounds from *Teucrium chamaedrys* L.. Nat. Prod. Res..

[16] Xue Z., Yang B. (2016). Phenylethanoid glycosides: Research advances in their phytochemistry, pharmacological activity and pharmacokinetics. Molecules.

[17] Haïdara K., Alachkar A., Moustafa A. (2011). *Teucrium polium* plant extract provokes significant cell death in human lung cancer cells. Health.

[18] Pacifico S., D’Abrosca B., Scognamiglio M., D’Angelo G., Gallicchio M., Galasso S., Monaco P., Fiorentino A. (2012). NMR-based metabolic profiling and in vitro antioxidant and hepatotoxic assessment of partially purified fractions from Golden germander (*Teucrium polium* L.) methanolic extract. Food Chem..

[19] Stanković M.S., Mitrović T.L., Matić I.Z., Topuzović M.D., Stamenković S.M. (2015). New values of *Teucrium* species: In vitro study of cytotoxic activities of secondary metabolites. Not. Bot. Horti Agrobot. Cluj Napoca.

[20] Özel Ş., Süntar İ., Ercan Gökay N., Taşkın Türkmenoğlu T., Demırel M.A. (2020). The effectiveness of *Teucrium chamaedrys* L. extracts on endometriotic implant regression in rat endometriosis model. Vet. Res. Forum.

[21] Croce A., Stinca A., Santangelo A., Esposito A. (2019). Exploring vascular flora diversity of two protected sandy coastal areas in southern Italy. Rend. Lincei. Sci. Fis. Naturali..

[22] Sumner L.W., Amberg A., Barrett D., Beale M.H., Beger R., Daykin C.A., Fan T.W., Fiehn O., Goodacre R., Griffin J.L. (2007). Proposed minimum reporting standards for chemical analysis Chemical Analysis Working Group (CAWG) Metabolomics Standards Initiative (MSI). Metabolomics.

[23] Pacifico S., Piccolella S., Marciano S., Galasso S., Nocera P., Piscopo V., Fiorentino A., Monaco P. (2014). LC-MS/MS profiling of a mastic leaf phenol enriched extract and its effects on H_2_O_2_ and Aβ(25–35) oxidative injury in SK-B-NE(C)-2 cells. J. Agric. Food Chem..

[24] Pacifico S., Piccolella S., Galasso S., Fiorentino A., Kretschmer N., Pan S.P., Bauer R., Monaco P. (2016). Influence of harvest season on chemical composition and bioactivity of wild rue plant hydroalcoholic extracts. Food Chem. Toxicol..

[25] Fiorentino A., D’Abrosca B., Pacifico S., Mastellone S., Piccolella S., Monaco P. (2007). Isolation, structure elucidation, and antioxidant evaluation of Cydonioside A, an unusual terpenoid from the fruits of *Cydonia vulgaris*. Chem. Biodivers..

[26] Pacifico S., Piccolella S., Lettieri A., Nocera P., Bollino F., Catauro M. (2017). A metabolic profiling approach to an Italian sage leaf extract (SoA541) defines its antioxidant and anti-acetylcholinesterase properties. J. Funct. Foods.

[27] Antognoni F., Iannello C., Mandrone M., Scognamiglio M., Fiorentino A., Giovannini P.P., Poli F. (2012). Elicited *Teucrium chamaedrys* cell cultures produce high amounts of teucrioside, but not the hepatotoxic neo-clerodane diterpenoids. Phytochemistry.

[28] Gross G.-A., Lahloub M.F., Anklin C., Schulten H.-R., Sticher O. (1988). Teucrioside, a phenylpropanoid glycoside from *Teucrium chamaedrys*. Phytochemistry.

[29] Scognamiglio M., Fiumano V., D’Abrosca B., Esposito A., Choi Y.H., Verpoorte R., Fiorentino A. (2014). Chemical interactions between plants in Mediterranean vegetation: The influence of selected plant extracts on *Aegilops geniculata* metabolome. Phytochemistry.

[30] Li R., Morris-Natschke S.L., Lee K.H. (2016). Clerodane diterpenes: Sources, structures, and biological activities. Nat. Prod. Rep..

[31] Fiorentino A., D’Abrosca B., Esposito A., Izzo A., Pascarella M.T., D’Angelo G., Monaco P. (2009). Potential allelopathic effect of neo-clerodane diterpenes from *Teucrium chamaedrys* (L.) on stenomediterranean and weed cosmopolitan species. Biochem. Syst. Ecol..

[32] Fiorentino A., D’Abrosca B., Ricci A., Pacifico S., Piccolella S., Monaco P. (2009). Structure determination of chamaedryosides A—C, three novel nor-neo-clerodane glucosides from *Teucrium chamaedrys* by NMR spectroscopy. Magn. Reson. Chem..

[33] Prior R.L., Hoang H., Gu L., Wu X., Bacchiocca M., Howard L., Hampsch-Woodill M., Huang D., Ou B., Jacob R. (2003). Assays for hydrophilic and lipophilic antioxidant capacity (Oxygen Radical Absorbance Capacity (ORACFL)) of plasma and other biological and food samples. J. Agric. Food Chem..

[34] Candela L., Formato M., Crescente G., Piccolella S., Pacifico S. (2020). Coumaroyl flavonol glycosides and more in marketed green teas: An intrinsic value beyond much-lauded catechins. Molecules.

[35] Elizondo G., Medina-Diaz L.M. (2003). Induction of CYP3A4 by 1α,25-dyhydroxyvitamin D3 in HepG2 cells. Life Sci..

